# Osteopontin: An important protein in the formation of kidney stones

**DOI:** 10.3389/fphar.2022.1036423

**Published:** 2022-11-09

**Authors:** Qingxia Jia, Ziye Huang, Guang Wang, Xia Sun, Yuyun Wu, Bowei Yang, Tongxin Yang, Jianhe Liu, Pei Li, Jiongming Li

**Affiliations:** ^1^ The Department of Urology, The Second Affiliated Hospital of Kunming Medical University, Kunming, China; ^2^ Department of Clinical Pharmacology, Xiangya Hospital, Central South University, Changsha, China

**Keywords:** kidney calculi, osteopontin, macrophages, T helper cells, dendritic cells, mast cells

## Abstract

The incidence of kidney stones averages 10%, and the recurrence rate of kidney stones is approximately 10% at 1 year, 35% at 5 years, 50% at 10 years, and 75% at 20 years. However, there is currently a lack of good medicines for the prevention and treatment of kidney stones. Osteopontin (OPN) is an important protein in kidney stone formation, but its role is controversial, with some studies suggesting that it inhibits stone formation, while other studies suggest that it can promote stone formation. OPN is a highly phosphorylated protein, and with the deepening of research, there is growing evidence that it promotes stone formation, and the phosphorylated protein is believed to have adhesion effect, promote stone aggregation and nucleation. In addition, OPN is closely related to immune cell infiltration, such as OPN as a pro-inflammatory factor, which can activate mast cells (degranulate to release various inflammatory factors), macrophages (differentiated into M1 macrophages), and T cells (differentiated into T1 cells) etc., and these inflammatory cells play a role in kidney damage and stone formation. In short, OPN mainly exists in the phosphorylated form in kidney stones, plays an important role in the formation of stones, and may be an important target for drug therapy of kidney stones.

## 1 Osteopontin

OPN was first discovered from the matrix of bovine bone. The protein consists of approximately 314 amino acids, contains a calcium-binding domain and multiple phosphate sites, its structural domain is rich in aspartate, glutamate, and serine residues, and has a molecular weight between 44 and 75 kDa (Franzén et al., 1985; Kläning et al., 2014). The molecular structure of OPN accounts for the largest proportion of phosphorylation sites, with more than 36 sites (Yalak and Vogel, 2012; Kariya et al., 2014). OPN is phosphorylated by Golgi-casein kinases, and the phosphorylation level and site are affected by the o-glycosylation state (OPN with five o-glycosylation sites deleted from threonine/proline rich regions increases cell adhesion activity and phosphorylation, inhibits integrin association) (Kariya et al., 2014) and 1,25-(OH)2D3 (induced upregulation of OPN mRNA expression, while promoting phosphorylated OPN converts to the non-phosphorylated state) (Safran et al., 1998). The degree of phosphorylation of OPN affects cell adhesion and interaction with other proteins (Kariya et al., 2021).

## 2 Effect of osteopontin on kidney stone formation

Whether OPN promotes or inhibits nephrolithiasis is currently under debate (Huang et al., 2022; Liu et al., 2022). An important process of kidney stone formation is the transformation of retained crystals in the renal tubules into “concrete” stones, of which calcium oxalate (CaOx) is the main component of kidney stones and OPN is the main component of the calcium-containing stone matrix in the kidney (Okada et al., 2008). As early as 2003, Konya et al. compared the effects of OPN, fibronectin, Tamm-Horsfall glycoprotein, vitronectin, and laminin on CaOx crystallization *in vitro*, and found that OPN immobilized on the surface of collagen particles enhanced the adhesion and aggregation of seeds, and the adhesion of newly formed crystals is enhanced (Konya et al., 2003).

In 2008, Okada’s team induced renal CaOx stone formation by intraperitoneal injection of 100 mg/kg glyoxylate into wild-type mice (WT) and OPN knockout mice (KO) for 1 week. The results showed that the number of crystals in WT was significantly higher than that in KO, and large flower-shaped crystals were seen in WT tubules, while KO showed small and uniform crystals. Immunohistochemical staining of OPN showed that WT renal crystals contained OPN protein but KO renal crystals did not (Okada et al., 2008). In 2013, Tsuji’s team used 1.5% ethylene glycol to induce CaOx nephrolithiasis in rats and found that the expression of OPN was increased in the kidney. Then using OPN siRNA transfected *in vivo* to knock down OPN, the results showed that the expression of OPN and renal crystals were significantly reduced in the knockdown group compared with the stone model group (Tsuji et al., 2014). However, in a study published in 2003, Wesson et al. induced significant CaOx crystals in the kidneys of KO mice but not WT mice after 4 weeks of induction with 1% ethylene glycol. And OPN immunohistochemistry showed significant expression in the kidneys of WT mice, from which they believed that OPN, as an inhibitor of CaOx crystal formation and renal tubular retention, played a key renoprotective role *in vivo* (Wesson et al., 2003). Interestingly, later experiments by Okada’s team found that 1%, 5%, and 10% ethylene glycol could not successfully induce the WT mice kidney CaOx stone model (Okada et al., 2007). This seems to suggest that the species used in the modeling of kidney stones, as well as the stone-inducing drugs and dosages, could lead to opposite conclusions. This may be the reason why there are still two views in recent years: inhibition (Paloian et al., 2016; Imig, 2022; Liu et al., 2022) and promotion (Li et al., 2019; Zhou et al., 2022a; Huang et al., 2022). Although the effect of OPN on renal calculi is controversial, we can see that OPN plays an important role in the formation of CaOx crystals.

## 3 Localization of osteopontin in the kidney

**FIGURE 1:**
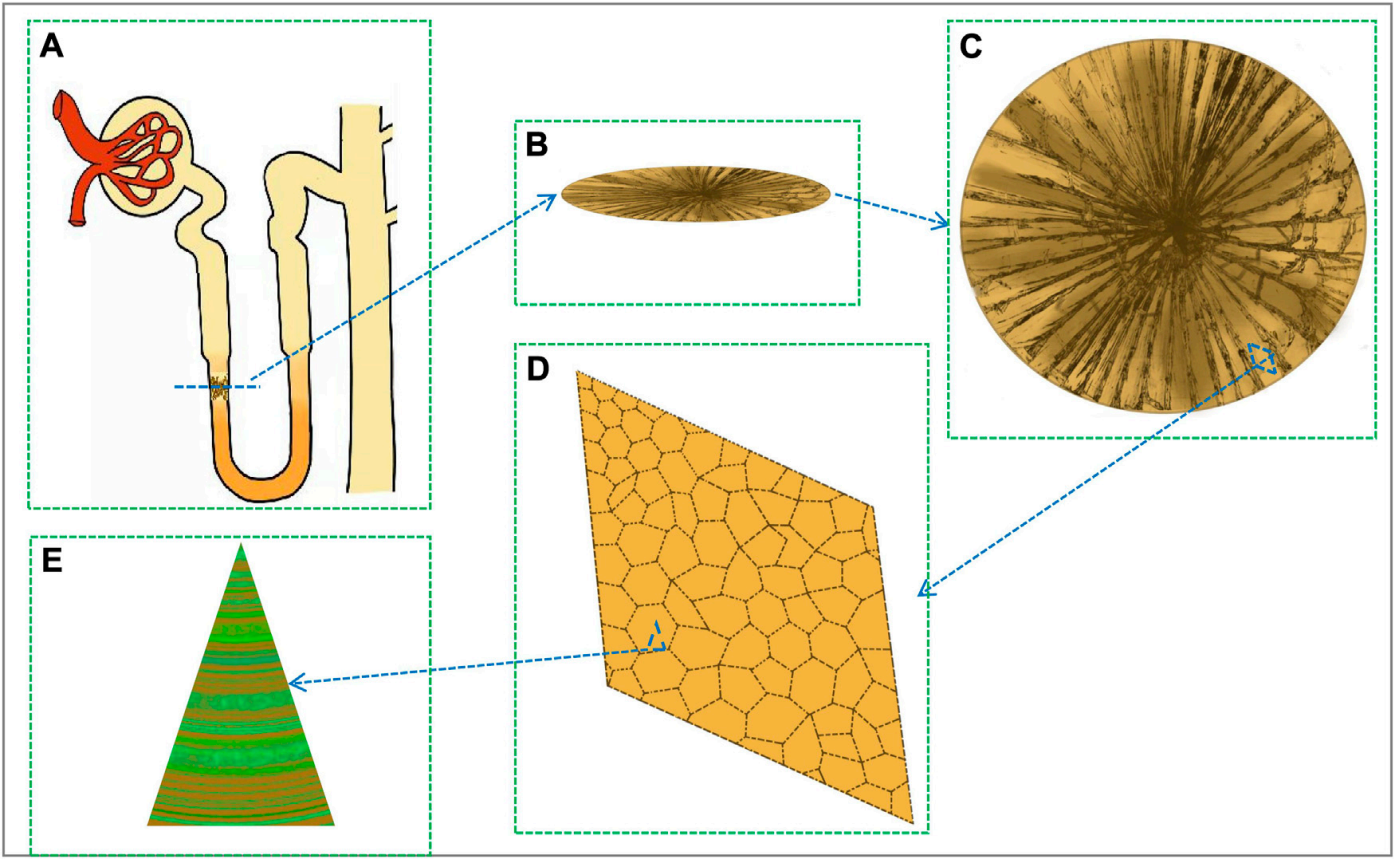
The location and crystal structure of stone crystals in the renal tubule. **(A)** Crystal deposition on the descending limb of Henle’s ring. **(B)** Horizontal section of the crystal. **(C)** Crystal front side. **(D)** Crystal enlarged structure. **(E)** OPN-crystal-bound complex (fluorescent green is OPN protein, yellow is crystal).

OPN is mainly present in the descending limb cells of Henle’s loop and in the papillary surface epithelium of the calyx fornix region, where it has a relatively rapid turnover and may be physiologically regulated (Imig, 2022). During hyperoxaluria, OPN expression is increased in the kidney, but remains predominantly restricted to fine limb and papillary surface epithelial cells. After deposition of CaOx crystals, OPN expression was observed in the entire kidney, including the proximal tubules (Paloian et al., 2016). OPN is localized to cells of Henle’s loop and collecting ducts as well as to plaque sites. Under the immunoelectron microscope, OPN mainly appeared on the surface of the apatite crystal phase, at the junction between the crystal and the organic layer (Paloian et al., 2016), and between the CaOx stones like annual rings (Li et al., 2019) (Figure 1).

## 4 Osteopontin induces the production of inflammatory immune cells in the kidney

### 4.1 Osteopontin induces monocyte/macrophage chemotaxis

The phenotype and function of macrophages are regulated by the surrounding microenvironment. It is well known that the equilibrium polarization of M1/M2 macrophages determines the fate of an organ in response to inflammation or injury. When infection or inflammation is severe, macrophages differentiate into M1 phenotype and release inflammatory factors (such as TNF-α, IL-12, and IL-23), while tissue damage will induce macrophages to differentiate into M2 type and secrete a large amount of IL-10 and TGF-β to inhibit inflammation and repair damage (Zhou et al., 2022a). OPN is a pro-inflammatory and monocyte chemokine (Kleinman et al., 1995). When kidney cell damage produces large amounts of OPN, it drives monocytes into the kidneys, prompting macrophages of monocytes to infiltrate and differentiate into the M1 pro-inflammatory phenotype, leading to renal fibrosis (Khan et al., 2002). Factors that contribute to kidney cell damage include hypertension [e.g., kidney damage after catecholamines and salt-sensitive hypertension (Johnson et al., 1999), hydronephrosis hypertension after ureteral obstruction (Yoo et al., 2006)], hypoxia [e.g., renal ischemia/reperfusion (Feitoza et al., 2008)], toxin (Pichler et al., 2008), infection, etc.

Kidney stones are epidemiologically and histopathologically associated with kidney disease and may contribute to chronic kidney disease and end-stage renal disease (Zisman et al., 2015); renal mononuclear phagocytes, especially macrophages, regulate crystal development (Zisman et al., 2015; Okada et al., 2010; Ichikawa et al., 2014). The renal CaOx crystals of hyperoxalate mice disappeared spontaneously and expressed various macrophage-related cytokines and chemokines (Okada et al., 2010). Urinary and renal tubular CaOx monohydrate crystals are broken down and dissolved in the presence of macrophages (Vervaet et al., 2009).

Populations of mononuclear phagocytes, including macrophages, have diverse responses in kidney disease (Williams et al., 2010). Several reports suggest that M2 macrophages have anti-inflammatory and tissue-healing effects *in vivo* models of nephropathy and ischemia/reperfusion acute kidney injury (Tang et al., 2020; Zhang H et al., 2021; Zheng F et al., 2021). However, pro-inflammatory M1 macrophages can worsen renal conditions, leading to chronic kidney disease and fibrosis (Wang et al., 2021). In addition, large numbers of M1 macrophages contribute to the development of renal crystal deposition in mice with metabolic syndrome (Taguchi et al., 2015). Induction of M2 macrophages by colony-stimulating factor (CSF)-1 in M2-deficient mice inhibited renal CaOx crystal formation (Taguchi et al., 2016). In hyperoxalate C57BL/6J mice, infusion of M1 macrophages and M1-inducing factors (LPS and IFN-γ) promoted renal crystal formation, while M2 macrophages and M2-inducing factors (IL-4 and IL-13) Infusion inhibited renal crystal formation. These M2 macrophage treatments decreased the expression of crystallographic-related genes such as OPN and CD44, whereas M1 macrophage treatment increased the expression of pro-inflammatory and adhesion-related genes such as IL-6, NOS, and TNF-α (Taguchi et al., 2016). This indicates that M2 macrophages inhibit the development of renal crystals, which may be related to the reduction of the stone-promoting factor OPN.

### 4.2 Osteopontin and T helper cells

There are two main types of helper T cells: Th1 and Th2 cells, which are inflammatory T cells and anti-inflammatory T cells, respectively (Rautajoki et al., 2008). OPN gene expression in activated T cells is regulated by T-bet, a transcription factor that controls CD4+ T helper (Th1) cell lineage commitment. T-bet-dependent expression of OPN in T cells is critical for efficient tilting of CD4+ T and CD8+ T cells towards the Th1 and type 1 CD8+ T cell pathways, respectively (Shinohara et al., 2005). In allergic asthma, OPN enhances sensitization and downregulates Th2-driven IL-4-dominant inflammation. Increased OPN expression suppresses Th2 effects during specific immunotherapy. In Th1-driven delayed-type hypersensitivity, such as allergic contact dermatitis, OPN supports dendritic cell (DC) migration and IL-12 expression and is secreted by T effector cells and keratinocytes, enhancing Th1-mediated hypersensitivity and supports the chronicity of disease (Frenzel and Weiss, 2011).

Studies have reported that in atherosclerosis, Th1 cells drive pro-inflammatory responses and Th2 drive anti-inflammatory responses, similar to urinary calculi, with similar calcified lesions on vascular endothelial cells (Hsi et al., 2016; Ley, 2020). Interestingly, the pro-inflammatory Th1 phenotype is mainly present between renal mesangial cells and renal tubular epithelial cells (Iwata et al., 2014). In the renal papillae containing the Randall’s plaques (thought to be the starting point of stone formation), the helper T-cell signaling pathway protein is upregulated. Thus, helper T-cell immune responses and associated inflammatory processes appear to lead to the formation of calcium phosphate stones on Randall’s plaques (Taguchi et al., 2019).

### 4.3 Osteopontin and dendritic cells

The mRNA of OPN is up-regulated in the early stage of DC progenitor cell differentiation, and a large amount of OPN is synthesized and secreted, and it is enhanced with stimulation (Kawamura et al., 2005; Blengio et al., 2013). Mature DC are activated by inflammatory or pathogenic factors to generate specific immune responses. Under the anti-inflammatory regulation of factors, DC play a role in regulating Treg differentiation, and the secreted OPN is regulated by these anti-inflammatory factor (Del Prete et al., 2019). DC migration and immune function are also regulated by OPN. OPN mediates DC migration by binding to DC-expressed CD44 and αvβ3 receptors (Weiss et al., 2001). OPN can activate DC-releasing factors (such as IL-12, IFN-γ and TNF-α) to induce the differentiation of Th cells to produce Th1 (Renkl et al., 2005), and may also regulate the balance of Th1/Th2 and limit the Th2 response (Kurokawa et al., 2009).

### 4.4 Osteopontin and mast cells

Mast cells (MC), the constituent cells of the kidney, are small in number but significantly increased in various kidney diseases. Studies in MC-deficient rats have shown that they have ameliorating effects on renal fibrosis (Miyazawa et al., 2004). In anti-glomerular basement membrane antibody-induced glomerulonephritis, MC protect against the damaging effects of glomerular injury by initiating repair and remodeling functions. Protection may also include limiting the immunomodulatory capacity of autoreactive T cell responses (Kanamaru et al., 2006). MC also control tubulointerstitial fibrosis by activating tissue remodeling and neutralizing fibrotic factors (Mouchet et al., 2021). However, mediators released by MC activation and degranulation during inflammation promote the destruction of renal architecture, leading to renal interstitial fibrosis (Summers et al., 2012; Kaltenecker et al., 2020; Zhou et al., 2022b). Therefore, the physiological environment in which other cells and inflammatory mediators interact determines the ultimate role of MC in the development of kidney disease (Bulfone-Paus and Paus, 2008).

OPN can bind to MC surface CD44, αv integrin receptor, and enhance IgE-mediated MC degranulation and migration (Bulfone-Paus and Paus, 2008). While other studies found that immobilized OPN enhanced the interaction with MC, soluble OPN did not have any obvious effect. OPN and integrin domains mediate activation of MC by immobilized OPN, but not CD44 on MC. OPN immobilized on the extracellular matrix can modulate human adaptive immunity by retaining MCs at sites of inflammation and inhibiting the release of anti-IgE-induced cytokines in MCs (Ng et al., 2018). There is now substantial evidence for the important role of MC in fibrotic diseases (Samitas et al., 2011). However, studies from different clinical settings and different animal models have drawn partially conflicting conclusions about how MC affects fibrosis, with many studies suggesting that MC has a deleterious effect, while others suggest that MC may play a protective role (Bradding and Pejler, 2018). However, it is certain that MC activation and degranulation promote renal interstitial fibrosis (Summers et al., 2012; Zhou et al., 2022b).

## 5 Osteopontin and drug therapy for kidney stones

### 5.1 CaOx stone formation process

The average incidence of kidney stones is 10%, and it is 20–25% in the Middle East. The incidence is different due to factors such as geography, climate, and dietary structure. The recurrence rate of kidney stones is about 10% in 1 year, 35% in 5 years, 50% in 10 years, and 75% in 20 years (Huang et al., 2022). There are five main mineralogy components of kidney stones, including CaOx, carbonapatite, urate, magnesium ammonium phosphate, and calcite (Kleeman et al., 1980; Ye et al., 2020). More than 80 percent of human kidney stones are CaOx and calcium phosphate stones, either alone or in admixture, which are calcium-opaque stones (Kleeman et al., 1980; Fink et al., 2012; Aggarwal et al., 2013). The pathological mechanism of nephrolithiasis is complex, and there are many etiologies. The general view is that the stones are formed on the basis of Randall’s plaque (calcified plaques formed on the surface of the renal papilla), and the stones start from the basement membrane of the thin limbs of the Henle’s ring on the surface of the renal papilla (Stoller et al., 1996; Sherer et al., 2018; Wiener et al., 2018; Khan et al., 2016; Khan et al., 2021). CaOx stones are usually attached to Randall’s plaques on the surface of the renal papilla, which are mainly composed of calcium phosphate crystals mixed with a protein-rich organic matrix (including the main ingredient OPN) (Khan et al., 2021). Randall’s plaques formation is associated with the presence of pro-inflammatory macrophages M1 and downregulation of anti-inflammatory macrophages M2 in the surrounding renal tissue (Taguchi et al., 2016). In animal models, crystal deposition in the kidney is associated with ROS production, inflammasome activation, and increased expression of inflammatory cascade-related molecules such as OPN (Umekawa et al., 2004; Li et al., 2014; Letavernier et al., 2018). Hyperoxaluria and CaOx stones induce ROS production and oxidative stress to promote kidney damage, and the subsequent inflammatory and immune responses lead to the formation of Randall’s plaques and calcium stones (Khan et al., 2021).

### 5.2 Research status of drug therapy for CaOx stones

Citrate is a drug widely used in clinical practice to prevent kidney stones. Its therapeutic mechanism is to reduce the formation of stones by increasing the citrate and pH in the urine (Barcelo et al., 1993). A meta-analysis of seven randomized controlled trials of oral citrate in the treatment of kidney stones included 477 patients, most of whom had oxalate stones. At 6, 12, and 24 months after treatment initiation, reduction in stone size (reduction or complete disappearance of residual debris) or reduction in stone recurrence using abdominal plain radiographs or intravenous urography or computed tomography (CT) scans (new stone formation). Four of its studies including 160 participants showed that oral citrate significantly reduced stone size. And seven studies including 324 patients showed significantly less new stone formation in the citrate-treated group. Four studies reported adverse events, mainly gastrointestinal symptoms (Such as loss of appetite, nausea, vomiting, and diarrhea). Compared with the control group, more patients in the citrate-treated group dropped out due to factors such as adverse events or intolerance (Phillips et al., 2015). A study published in *Nature* in 2016 compared the effect of hydroxycitrate and citrate in inhibiting CaOx crystals from the molecular structure level. The results showed that both hydroxy citrate and citrate have the ability to dissolve and inhibit the formation of CaOx crystals. The effect of hydroxycitrate is better (Chung et al., 2016). *In vitro* studies have shown that hydroxycitrate has a calcium-binding capacity comparable to that of citrate, and it is an effective inhibitor of calcium oxalate monohydrate crystallization (Kim et al., 2019), and *in vivo* studies have shown that hydroxycitrate has a stone-dissolving effect better than citrate (Yang et al., 2022). Hydroxycitrate, a structural analog of citrate, is currently used primarily as an over-the-counter supplement for weight loss (Márquez et al., 2012). Clinical studies have shown that oral hydroxycitrate also has more gastrointestinal symptoms, and most patients cannot tolerate long-term use (Heymsfield et al., 1998).

### 5.3 Nephrolithiasis treatment drugs can significantly inhibit the expression of osteopontin

Citrate is currently the main oral drug for preventing stones, and it is also a litholytic drug recommended by the guideline (Skolarikos et al., 2021). The rat experiment found that after citrate treatment, the reduction of stones was accompanied by a significant reduction in OPN and inflammation (Yasui et al., 2001; Chen et al., 2013; Alex et al., 2014). Hydroxycitrate, which is more effective than citrate, also significantly inhibited OPN expression, as well as reduced oxidative stress and inflammation to inhibit renal CaOx deposition (Liu et al., 2020). A positive association between OPN and stone reduction has also been shown in other studies of drugs [such as astaxanthin (Alex et al., 2014), resveratrol (Qin et al., 2018), and gallotannin (Lee et al., 2012)] for the treatment of nephrolithiasis. This means that drugs that can significantly inhibit the expression of OPN have a therapeutic effect on kidney stones.

## 6 Conclusion

**FIGURE 2:**
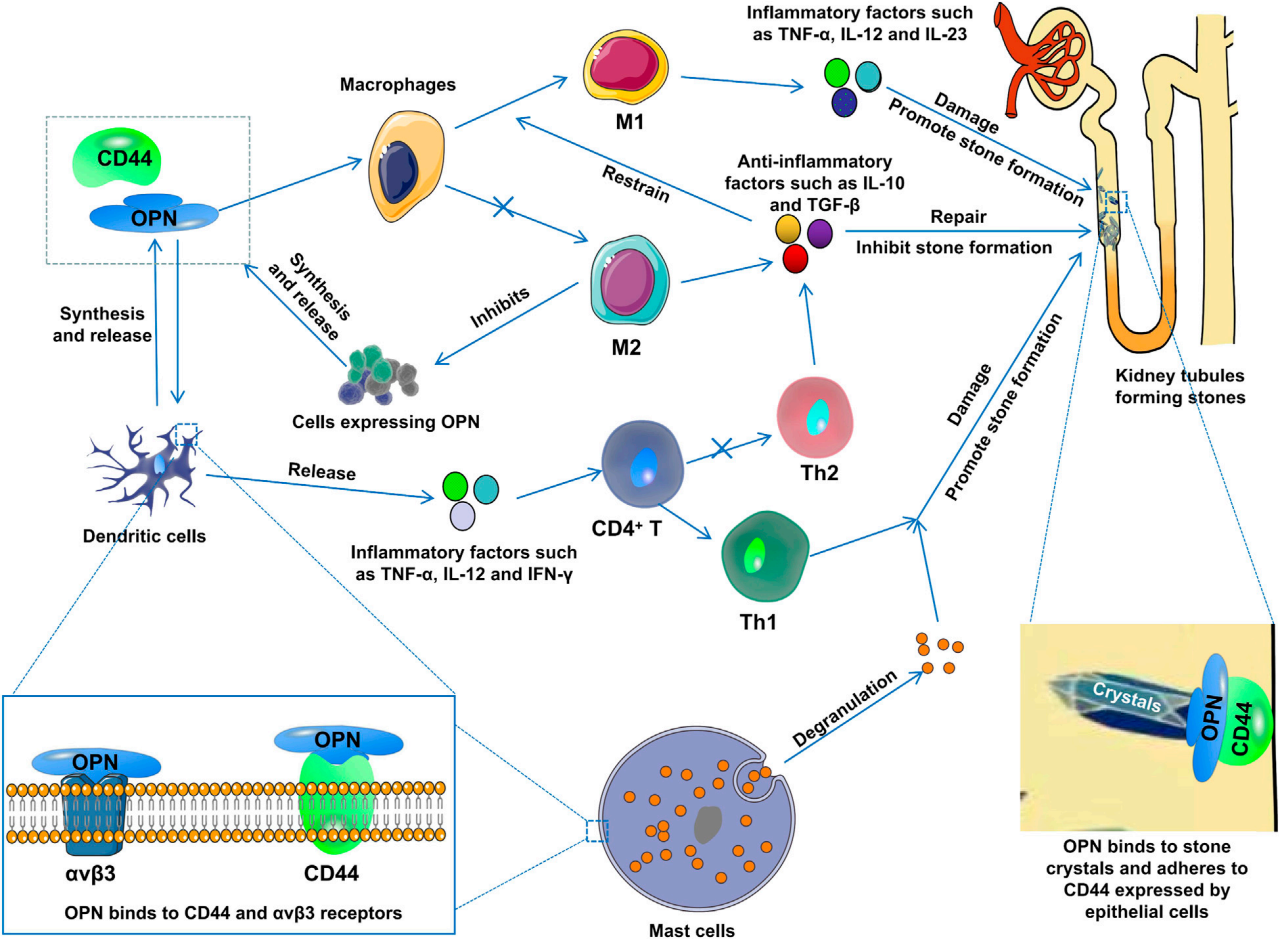
OPN induces macrophages to differentiate into M1 macrophages, induces T cells to differentiate into T1 cells, and activates mast cell degranulation to release various inflammatory mediators, which damage kidneys and promote stone formation.

The role of OPN in kidney stone formation is controversial, with some studies suggesting that it inhibits stone formation, while other studies suggest that it can promote stone formation. As research progresses, there is growing evidence that it promotes stone formation. OPN is a highly phosphorylated protein, and some studies have shown that the phosphorylated protein has an adhesive effect, promoting stone aggregation and nucleation. However, other studies suggest that it prevents stone aggregation and nucleation. In addition, OPN is closely related to immune cell infiltration, such as OPN as a pro-inflammatory factor, which can activate mast cells (degranulate to release various inflammatory mediators), macrophages (differentiated into M1 macrophages), T cells (differentiated into T1 cells), and these inflammatory cells play a role in damaging the kidneys and promoting stone formation (Figure 2). In conclusion, OPN mainly exists in the phosphorylated form in kidney stones, plays an important role in the formation of stones, and may be an important target for drug therapy of kidney stones.
